# Prevalence and Predictors Associated to *Schistosoma mansoni* Infection among Patients Attending the Saint Jean de Malte Hospital, Njombe, Littoral Region, Cameroon

**DOI:** 10.1155/2023/8674934

**Published:** 2023-10-21

**Authors:** Ambe Fabrice Ngwa, Pride Tanyi Bobga, Ekwi Damian Nsongmayi, Gedeon Schadrack Yememe Yememe, Judith Ngong Nyeme, Mohamed Isah, Ebai Christabel Ashu, Calvin Bisong Ebai

**Affiliations:** ^1^Department of Microbiology and Parasitology, Faculty of Science, University of Buea, Cameroon; ^2^Department of Medical Laboratory Sciences, Faculty Health Sciences, University of Buea, Cameroon; ^3^International School for Nurses and Technico-Sanitary Personnels, Douala, Cameroon; ^4^School of Medical and Biomedical Sciences, Fomic Polytechnic University, PO Box 123, Buea, Cameroon; ^5^Department of Public Health, Faculty of Medicine and Pharmaceutical Science, University of Dschang, Cameroon; ^6^Department of Public Health, Faculty of Medicine and Pharmaceutical Science, University of Douala, Cameroon; ^7^Department of Medical Laboratory Sciences, Faculty Health Sciences, University of Bamenda, Cameroon

## Abstract

**Background:**

Schistosomiasis remains a major public health concern in sub-Saharan Africa. It has been associated to morbidity and mortality in developing countries including Cameroon, and Njombe-Penja health district is an endemic area. This study is aimed at determining the prevalence and risk factors of *Schistosoma mansoni* infection among patients attending the Saint Jean de Malte Hospital, Njombe.

**Methods:**

A cross-sectional study design was employed, with the enrolment of 300 participants using convenience sampling technique. Data were collected using a structured questionnaire. Stool specimens were collected and examined using direct microscopy, Kato-Katz's method, and formol ether concentration technique. Data were analyzed using SPSS, and chi-square test was used to assess the association. Risk factors for *S. mansoni* infection were assessed using multivariable logistic regression, and a *p* < 0.05 was considered significant.

**Results:**

The overall prevalence of *Schistosoma mansoni* infection was 13%. *Schistosoma mansoni* infection was mostly frequent among patients < 20 years and males. Stream usage (AOR = 2.15, 95% CI. 1.32–3.50), always visiting the stream (AOR = 11.35, 95% CI 2.33-55.33), always swimming and washing clothes in the stream (AOR = 7 : 10, 95% CI 2.31-21.80), age group < 20 years (AOR = 3.7, 95% CI 1.1-12.2), and age group 20-29 years (AOR = 2.58, 95% CI 1.14-18.42) were significantly associated with increased risk of *S. mansoni* infection.

**Conclusion:**

These findings suggest that *Schistosoma mansoni* infection is of public health concern in Njombe and its environs. Age of <20 years and between 20 and 29 years, stream usage, always visiting the stream, and always swimming and washing clothes in the stream were the main risk factors of *S. mansoni* infection. Thus, mass drug administration and health education are required.

## 1. Introduction

### 1.1. Background

In sub-Saharan Africa (SSA), schistosomiasis is a major public health concern. *S. mansoni* is responsible for more than one-third of the 192 million cases of schistosomiasis that occur each year and is the causative agent of intestinal schistosomiasis [1]. About 4.4 million people infected with *S. mansoni* suffer from bloody diarrhoea, while hepatomegaly linked to periportal fibrosis, portal hypertension, and haematemesis affects approximately 8.5 million individuals [2]. Additionally, according to WHO's 2020 report, the disease's complications are thought to be the cause of 130,000 deaths annually. Chronic infections with *S. mansoni* in school-aged children (ages 6 to 15 years) result in anaemia, growth retardation, and cognitive impairment [2, 3]. The main morbidities, due to *S. mansoni* infection in preschool-aged children, under 6 years, are anaemia, hepatosplenomegaly, and hepatic fibrosis with a higher risk among children between 37 and 60 months [4]. The infection is more prevalent in most developing nations in Africa, Asia, and South America which are characterized with impoverished people without access to potable water and basic sanitation. The vast majority of cases (85%) occur in Africa. Schistosomiasis is the parasitic disease with the second-largest economic burden in tropical regions, after malaria [1, 5].

Schistosomiasis is more widespread in poor rural communities, particularly in places where fishing and agricultural activities are dominant [6]. People have been reported to be more susceptible to schistosome infections when engaging in water contact activities like swimming, bathing, obtaining water, and irrigated farming; male children are reported to be at higher risk of infection than female children [6]. Avoiding contact with contaminated freshwater can help prevent schistosomiasis, and better access to water, sanitation, and hygiene (WASH), information, education, and communication can help lower the risk of infection (IEC) [7].

Schistosomiasis is a severe public health issue in Cameroon since it poses a risk to more than 5 million people. Cameroon unveiled its initial strategic plan to fight schistosomiasis in 2004. Over time, significant progress has been accomplished. Although widespread treatment has reduced schistosomiasis prevalence by 70%, there are still some hotspots, such as Njombe-Penja, where transmission persists (PNLSHI, 2020). Findings from Njombe revealed that the population has been infected by *Schistosoma mansoni* with a prevalence of 13.07% [8] and 19.8% [9]. Also, Tonga et al. [10] reported the prevalence of 30% of *Schistosoma mansoni* infection among pregnant women in Njombe-Penja District, and the prevalence of 9.7% of schistosomiasis among school-aged children was reported in Njombe by Nzenou et al. [11]. Moreover, the endemicity of schistosomiasis in Njombe is linked to an equatorial climate that provides conditions suitable for the presence of the molluscs that encourage the transmission of this disease [12]. More so, the population uses streams for bathing, washing clothes, and household utensils, and these streams are suitable habitats for the Bulinus species intermediate host and thus constitute the main transmission source [12].

The national control program against schistosomiasis in Cameroon prioritizes mass drug administration of praziquantel to school-aged children, while the WHO strategy recommends reaching both school-based and community-based programs [13]. However, this approach in Cameroon prohibits all groups that require treatment from receiving effective preventative chemotherapy (PC) [14]. Adults, particularly members of high-risk groups like fishermen, irrigation workers, and women, were not treated during deworming programs because the donated medications were primarily intended for school-aged children [14]. Also, preventive chemotherapy (PC) coverage has been impacted by increased migration to this region (Njombe and its environs) as a result of insecurity and violence in the North-West and South-West Regions and the fact that preschool children are not yet targeted for schistosomiasis [15, 16]. More so, praziquantel-based control programs have only a temporary effect on transmission and are limited in their potential to interrupt disease transmission in the long term [17]. This above shortcoming has affected the achievement of schistosomiasis elimination. In view of these limitations and to identify the mechanisms for transmission and maintenance of this disease in Njombe-Penja, we planned to assess the prevalence and factors associated with *Schistosoma mansoni* infection among patients attending the Saint Jean de Malte Hospital, Njombe.

## 2. Materials and Methods

### 2.1. Study Design

This study was a hospital-based cross-sectional survey carried out between 6 October 2022 and 28 March 2023. At enrolment, an informed consent and assent form were obtained, and each study participant was asked to complete a questionnaire which consisted of sociodemographic and personal details, history of present illness, and water contact behaviours.

### 2.2. Study Area

This study was conducted in Saint Jean de Malte Hospital, Njombe, which received about 4000 patients annually, which is located in Njombe-Penja, Mungo Division, Littoral Region, Cameroon. Njombe is a cosmopolitan rural area with most inhabitants being farmers.

### 2.3. Study Population

All patients of both sexes and all age groups were eligible in this study and participants with the history of abdominal pain. Also, participants, who did not have any history of taking anthelmintic drug during the data collection or within the last three months, were included in the study.

### 2.4. Ethical Considerations

Ethical review and clearance were sought from the Institutional Review Board (IRB/FHS UB) and administrative clearance from the regional delegation of public health and from the director of the Saint Jean de Malte Hospital, Njombe.

### 2.5. Sample Size

The sample size is estimated using the Lorentz formula as calculated below:

(1) $$\begin{array}{c} n=\frac{\text{z}2p\left(1-p\right)\;}{e2\;} \end{array}$$

where *z* = 1.96; *p* is the prevalence of intestinal schistosomiasis (31%) obtained from a study carried out by Tonga et al. [10], in Njombe-Penja health district; and *e* is the error rate (0.05%). Sample size attained was 300.

### 2.6. Data Collection

#### 2.6.1. Administration of Questionnaires

The selected participants or participants' guardians were given a structured questionnaire after giving their informed consent. Following that, information about the participant's sociodemographic characteristics, knowledge of *Schistosoma mansoni* infection, and water contact behaviour was collected. Water contact was assessed by inquiring about stream usage, activities at streams (swimming and chores), frequency to stream per day, and open defecation.

#### 2.6.2. Sample Collection

The participants were instructed to collect a stool sample into a clean and wide mouth-labeled specimen container making sure that soil or other contaminants do not get into the stool container, closed firmly, and brought to the examination area as soon as possible (at most 15 minutes, especially for watery stool, so as to increase the chances of identifying trophozoite of parasites). Direct microscopy was done immediately, and 10% formalin was added into the rest of the stool specimen, making sure that the stool specimen was completely covered by formalin.

#### 2.6.3. Stool Analysis (Direct Microscopy)

Direct wet mount involves microscopic examination of fresh fecal specimens by wet preparations with physiological saline (saline wet mount) or iodine solution (iodine wet mount). The procedure provides rapid diagnosis for intestinal parasites when they are present in sufficient density in the fecal sample. The stool specimen was mixed for homogeneity, and a little quantity collected from the sample bottle was mounted in normal saline, covered with a cover slide, and examined for parasites using the 10x objective lens of a microscope. Parasites seen were identified by adding iodine to the preparation and using the 40x objective lens to observe and identify.

### 2.7. Formol Ether Concentration Technique (FEC)

A small amount of stool (about 3 g) was diluted in 7 ml of a 10% salt formalin solution before being sieved in a centrifuge tube to eliminate big debris and having 3 ml of ether added. The mixture was rapidly agitated for 30 s and then centrifuged for 2 min at 2000 rpm. Then, the supernatant was discarded, and the pellet was plated, using a pipette, between a slide and coverslip with Lugol and read under a light microscope using 10x and 40x objectives.

### 2.8. Kato-Katz's Technique

One Kato-Katz's thick smear was prepared from each stool sample using a template holding 41.7 mg of stool samples. They were mounted on slides and covered with malachite green-saturated cellophane. The slides were left for 24 h to clear for easy visualization of *S. mansoni* eggs before being examined under a microscope using 10x and 40x. The Kato-Katz thick technique was used as a confirmation test for *Schistosoma mansoni* infection as recommended by the WHO.

### 2.9. Data Management and Analysis

Questionnaires were designed using Epi Info version 7. Data were entered into Microsoft Excel and analyzed using Statistical Package for the Social Sciences (SPSS) version 20 for Windows. Data were collected using a questionnaire and checked for errors. Data were edited, coded, and entered into a computer using Microsoft Excel software. Backup storage was done in CD, and all raw data were stored safely in a computer secured with a password. The significance of the difference in prevalence with respect to sociodemographic factors was explored using Pearson's chi-square test. A *p* value of less than 0.05 was considered statistically significant.

## 3. Results

### 3.1. Sociodemographic Characteristics of Study Participants in Saint Jean de Malte Hospital, Njombe

Three hundred (300) patients aged ranging from 12 to 73 years were involved in this study. The mean age of the participants was 28.2 ± SD (8.56), and the median age was 26 years. The majority (44.3%) of the participants fall within the age group < 20 years, and the age group between 30 and 39 years constituted 15.7%. More than half (54%) of the participants were not married. The majority (38.7%) of the participants ended their education in secondary school, 36.7% in primary school, and 24.6% in the university. The majority (46.7%) of the participants were students (Table 1).

### 3.2. Prevalence of *Schistosoma mansoni* Infection among Patients Attending Saint Jean de Malte Hospital, Njombe

**Figure 1:**
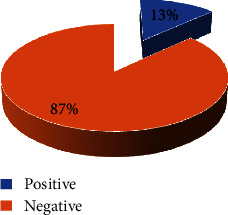
Prevalence of *Schistosoma mansoni* infection among patients attending the Saint Jean de Malte Hospital, Njombe.

Out of the 300 patients screened for *Schistosoma mansoni* infection, 39 of our study population were positive, giving the prevalence of 13% as shown in Figure 1.

### 3.3. The Association between Sociodemographic Factors and *Schistosoma mansoni* Infection

The frequency of *Schistosoma mansoni* infection was highest among individuals in the age group < 20 years (Table 2). However, there was a significant association between infection rate with age (*p* = 0.009). *Schistosoma mansoni* infection was observed more frequently among farmers (5.3%) (Table 2). The observation between occupation and the prevalence of intestinal schistosomiasis was statistically significant (*p* < 0.001) (Table 2). *Schistosoma mansoni* infection was predominance among males (8.0%) than in females (5.0%) (Table 2). The prevalence of *Schistosoma mansoni* infection and gender was significantly associated (*p* = 0.002). There was a significant association (*p* < 0.001) between educational level and the prevalence of *Schistosoma mansoni* infection. There was a high infection rate (7%) among participants that ended at primary school level.

### 3.4. Factors Associated with Prevalence of *S. mansoni* Infection

In univariable analysis, stream usage, always visiting the stream, always swimming or washing clothes in the stream, agegroup < 20years, and age group 20-29 years were significantly associated with *S. mansoni* infection, while the remaining variables were not observed to have any significant association with *S. mansoni* infection. In multivariate analysis, the odds of positive *S. mansoni* infection were significantly four times higher among individuals less than 20 years (AOR = 3.7, 95% CI 1.1-12.2, *p* = 0.03), and participants that were between 20 and 29 years were twice positively associated with *S. mansoni* infection (Table 3). The likelihood of *S. mansoni* (AOR = 2.58, 95% CI 1.14-18.42, *p* = 0.032) infection among participants who used stream was significant and about two times higher (AOR = 2.15, 95% CI. 1.32-3.50, *p* = 0.001). The odds of *S. mansoni* infection was also significantly eleven times higher in those that frequently used the stream compared to never used the streams at all (AOR = 11.35, 95% CI 2.33-55.33, *p* = 0.003). Subjects who swim and wash clothes in streams were seven times positively associated with *S. mansoni* infection (AOR = 7 : 10, 95% CI 2.31-21.80, *p* = 0.001) (Table 3).

## 4. Discussion, Conclusions, and Recommendations

### 4.1. Discussion

This study was designed to determine the prevalence of *Schistosoma mansoni* and risk factors associated with patients attending Saint Jean de Malte Hospital, Njombe. The overall prevalence of *Schistosoma mansoni* infection was 13%. Our result is less than 19.8% prevalence of intestinal schistosomiasis reported in Njombe-Penja [9]. The decreased prevalence of intestinal schistosomiasis might be due to regular annual mass praziquantel treatment at the study site. Also, strictly following such important preventive chemotherapy in the area of high transmission for schistosomiasis may have a positive impact on the prevalence and intensity of the disease and decrease stream contact. Other factors that might have contributed to the observed low prevalence of *Schistosoma mansoni* infection as reported by previous studies include the presence of a clean and safe water supply, good sanitation and hygiene, and good knowledge about the mode of transmission and prevention of the disease [2, 18].

Findings from the study demonstrated that *Schistosoma mansoni* infection was significantly more common in males. This may be explained by increased propensities for water interaction through swimming, playing, and other domestic activities like collecting water and doing laundry in polluted bodies of water. Our findings are probably because in semiurban areas, when children get older, the males take on harder domestic chores like collecting water and doing laundry and find pleasure in swimming in the stream for longer periods than the females. This higher prevalence of schistosomiasis among males is in line with other studies performed elsewhere [18]. These findings are, however, contradictory to that of Cisse et al. [2], which reported an association between the female sex and the prevalence of *S. mansoni* infection, and Ntonifor et al. [19], in the Mount Cameroon area in which the infection was more common in females.

*Schistosoma mansoni* infection was more common in patients less than 20 years, and there was a significant association between age and infection rate of *Schistosoma mansoni*. Patients less than 20 years and those 20-29 years were associated with higher odds of *S. mansoni* infection. The findings are in agreement with Njunda et al. [5], who reported a higher prevalence of schistosomiasis in children 10 years and below, which could be attributed to the different behavioural pattern and cultural practices of the different study populations. Also, patients of these age groups are predisposed to schistosomiasis due to their active life, hence increased water contact activities [6, 20]. Children are the most afflicted group of people in endemic areas, which considerably increases the risk of aquatic environment pollution. The declining trend in infection with ageing is caused by age-acquired immunity against reinfection. Also, it is possible that the older participants were applying what they knew about schistosomiasis, particularly with regard to the various preventive methods for effective schistosomiasis prevention and control. The finding is contrary to Payne et al. [9], where participants from 23 years and above were mostly infected with *S mansoni*.

There was an association between occupation and the prevalence of *Schistosoma mansoni* infection, and the odds of *Schistosoma mansoni* infection was significantly higher among farmers. Our results may be due to farming activities in swampy areas, hygienic conditions, and low educational knowledge about *Schistosoma mansoni* infection. The education level of the inhabitants was also strongly associated with the transmission of *S. mansoni*, and the odds of infection was higher among primary school participants. This is probably because education turns to improve knowledge about schistosomiasis. Participants who are mostly on the course of secondary education can carry out more independent reading and quest for more information if the need arises than their counterparts, thus leading to improve knowledge. Our result is in agreement with Payne et al. [9], Green et al. [6], and Wepnje et al. [12], where education was significantly associated with *Schistosoma mansoni* infection.

In this study, stream usage, visiting stream, and swimming or washing clothes in the stream were identified as the associated factors for infection with *Schistosoma mansoni*. Many of the patients that come to this hospital are from Njombe, Penja, and other rural areas, where there is little access to water for bathing and household usage, forcing residents to utilise streams and rivers instead, which puts them at risk of contracting schistosomiasis. The high dependence of the population on rivers and streams avails each water contact activity as a potential risk factor of *S. mansoni* infection. This confirms previous reports in other endemic foci in Cameroon [12, 18] and elsewhere [20, 21]. Water contact at any point in time is linked mostly to practices including domestic activities and bathing. The activities that expose people to cercaria-infested water the most are laundry, bathing, playing, and recreational swimming since they necessitate submerging large amounts of body parts for prolonged periods of time [2, 22]. Hence, avoiding domestic and recreational activities such as swimming or fishing in infested water may curb the spread of the disease.

## 5. Conclusions

These findings suggest that *Schistosoma mansoni* infection is of public health concern in Njombe, and the prevalence of 13% was reported. The burden of infection is higher in males, farmers, and participants < 20 years with most of the participants having a poor knowledge about the disease. Stream usage, always visiting the stream, always swimming or washing clothes in the stream, age group < 20 years, and age group 20-29 years were significantly associated with *S. mansoni* infection. These findings stress the need to include all age groups in the mass drug administration (MDA) program with praziquantel. However, control measures to effectively control and eventually eliminate the infection in this area will entail the provision of a safe and adequate water supply, health education, and snail control.

## Figures and Tables

**Table 1:** Sociodemographic characteristics of study participants in Saint Jean de Malte Hospital, Njombe.

Variable	Category	Frequency	Percentages
Age		Mean age: 28.2 ± SD (6.56)	
	<20	133	44.3
	20-29	53	17.7
	30-39	47	15.7
	≥40	67	22.3

Occupation	Student	140	46.7
	Teacher	57	19.0
	Farmer	65	21.7
	Driver	33	11.0
	Business	5	1.6

Gender	Male	132	44.0
	Female	168	56.0

Education	Primary	110	36.7
	Secondary	116	38.7
	University	74	24.6

Marital status	Single	162	54.0
	Married	138	46.0

Religion	Christian	228	76.0
	Muslim	72	24.0

Total		300	100

**Table 2:** The prevalence of *Schistosoma mansoni* infection with respect to sociodemographic factors.

Variable	Category	Prevalence of intestinal schistosomiasis, *n* (%)	Chi-square	*p* value
Age	<20	18 (6.0)	11.456	0.009
	20-29	13 (4.3)		
	30-39	5 (1.7)		
	≥40	3 (1.0)		
	Total	39 (13)		

Occupation	Student	15 (5.0)	8.025	0.005
	Teacher	4 (1.3)		
	Farmer	16 (5.3)		
	Driver	3 (1.0)		
	Business	1(0.4)		
	Total	39 (13)		

Gender	Male	24 (8)	3.216	0.002
	Female	15 (5.0)		
	Total	39 (13)		

Education	Primary	21 (7)	30.799	<0.001
	Secondary	6 (2.0)		
	University	12 (4.0)		
	Total	39 (13)		

Marital status	Single	24 (8.0)	1.033	0.309
	Married	15 (5.0)		
	Total	39 (13)		

Religion	Christian	27 (9.0)	0.535	0.464
	Muslim	12 (4.0)		

Total	Total	39 (13)		

**Table 3:** Factors associated with the prevalence of *S. mansoni* infection.

Variables	COR (95% CI)	*p* value	AOR (95% CI)	*p* value
Age				
<20	3.8 (1.8-4.5)	0.042	3.7 (1.1-12.2)	0.03
20–29	2.18 (1.2-3.8)	0.023	2.58 (1.14-18.42)	0.032
30–39	2.06 (0.86-4.93)	0.103	2.5 (0.8-8.2)	0.13
≥40	1		1	
Gender				
Female	1		1	
Male	2.04 (1.08-3.85)	0.029	1.48 (0.574-3.84)	0.416
Level of education				
University	1		1	
Secondary	1.79 (0.91-3.92)	0.087	1.44 (0.64-3.22)	0.380
Primary	1.99 (0.83-3.46)	0.145	1.82 (0.55-2.69)	0.628
Stream usage				
No	1		1	
Yes	1.92 (1.25-2.94)	0.003	2.15 (1.32-3.50)	0.002
Frequency to stream per day				
Hardly	1		1	
Always	9.26 (3.04-28.17)	0.001	11.35 (2.33-55.33)	0.003
Some times	1.10 (0.36-3.36)	0.870	1.48 (0.33-6.70)	0.613
Activity at stream				
Washing clothes	1		1	
Swimming	2.72 (1.13-6.59)	0.026	2.50 (0.71-8.87)	0.156
Both	7.62 (3.48-16.68)	0.001	7.10 (2.31-21.80)	0.001
Open defecation				
No	1		1	
Yes	3.79 (1.73-8.30)	0.001	2.39 (0.41-13.90)	0.333
Occupation				
Driver	1		1	
Student	1.26 (0.63-2.50)	0.461	1.47 (0.64-3.23)	0.360
Farmer	2.07 (1.41-4.25)	0.042	2.57 (0.96-7.30)	0.094
Teacher	0.76 (0.58-2.73)	0.33	0.91 (0.67-4.36)	0.260
Business	—		—	

## Data Availability

The data will be made available upon request.

## Abbreviations

AOR: Adjusted odd ratio
CI: Confident intervals
COR: Crude odd ratio
FEC: Formol ether concentration
MDA: Mass drug administration
OR: Odd ratio
POC-CCA: Point-of-care
PSAC: Preschool-aged children
SAC: School-aged children
SSA: Sub-Saharan Africa
WHO: World Health Organization.
